# 

**Published:** 1903-01-03

**Authors:** 


**Notices of Births, Deaths, and Marriages** are inserted in this column at a charge of 2s. 6d. for an announcement not exceeding four lines.

### MARRIAGE.

ALPIN—STODDART.—At Mussooree, India, November 13th, by special license, Major W. Alpin, M.D., M.R.C.S., L.R.C.P., Indian Medical Service, to Helen May, eldest daughter of Colonel C. H. Stoddart, I.S.C., of Guernsey. (202)

### NOTICE TO CORRESPONDENTS.

All MSS., Letters, Books for Review, and other matters intended for the Editor should be addressed THE EDITOR, "THE HOSPITAL," THE HOSPITAL BUILDING, 28 & 29 SOUTHAMPTON STREET, STRAND, W.C.

The Editor cannot undertake to return rejected MS., even when accompanied by stamped directed envelope.

**Notice to Advertisers and Subscribers.**

All Advertisements, Orders for Copies of THE HOSPITAL, and Business Communications should be addressed to **THE MANAGER, (Not to the Editor)**, THE HOSPITAL, 28 & 29 Southampton Street, Strand, London, W.C.

The Cost of Subscription, post free, is as follows.—Per week, 2½d.; per quarter, 2s. 8d.; per half-year, 5s. 3d.; per annum, 10s. 6d. Foreign:—Per annum, 16s. 2d., payable, in all cases, in advance.

**NOTICE.—Vol. XXXII. of "The Hospital" (April to September 1902) in handsome cloth binding is now on sale at the office of this Journal, price, 7s. 6d. Cases for binding the Numbers contained in this Volume 1s. 6d. Index to Vol. XXXII., 2½d., post free.**

**The Monthly Part for January is now ready. Price 6d., by post 8d.**

### BOOKS RECEIVED.

**Baillière, Tindall and Cox.**

"Practical Points in Gynæcology." By H. Macnaughton-Jones, M.D.

"Report of the Chief Health Officer, Department of Public Health, New Zealand, 1902."

**A. and C. Black.**

"Who's Who, 1903."

"The Englishwoman's Year-book, 1903."

**J. Wright and Co., Bristol.**

"Our Baby." Eighth edition. By Mrs. Langton Hewer.

**Chatto and Windus.**

"Herbert Fry's Royal Guide to the London Charities, 1903."

### THE STUDENT OF MEDICINE.

**APPOINTMENTS.**

**Harold Austen**, M.D., B.S.Lond., M.R.C.S., L.D.S., appointed Assistant Dental Surgeon to St. Bartholomew's Hospital. **Miss C. A. Bennet**, M.B., Ch.B.Dub., appointed Resident Medical Officer of Dundee East Poorhouse and Hospital. **Alec. H. Brewer**, jun., M.R.C.S.Eng., L.R.C.P.Lond., appointed Honorary Anæsthetist to the German Hospital, Dalston. **H. J. Curtis**, B S. and M.D.Lond., F.R.C S.Eng., appointed Surgeon to the North-Eastern Hospital for Children, Hackney Road, N.E. **J. Malcolm Farquharson**, M.B., M.R.C.P.Edin., appointed Surgeon in charge of the Throat Department of the Eye, Ear, and Throat Infirmary, of Edinburgh. **John Hay**, M.D.Vict., M.R C.S., L.R C.P., appointed Honorary Assistant Physician to the Hospital for Consumption and Diseases of the Chest, Liverpool. **Robert V. Kelly**, C.B, F.R.C.S., L.R.C.P.Edin., appointed Government Medical Officer and Vaccinator at Delegate, New South Wales. **Henry R. Oswald**, M.D. C.M.Edin., appointed Coroner for the South-Eastern Division of London. **Charles Pollard**, F.R.C.S.Eng., appointed Honorary Surgeon to Worcester General Infirmary.

**"The Hospital" Institutional Library.**

**"Hospitals and Asylums of the World." 4 Vols., with a Portfolio of Plans, complete £12 12s.**

**"Burdett's Hospitals and Charities, 1902." 5s.**

**"Cottage Hospitals, General, Fever, and Convalescent." 10s. 6d.**

**"Hospital Expenditure: The Commissariat." 2s. 6d.**

**"A Legal Handbook for the Use of Hospital Authorities." 2s. 6d.**

**"The Cottage Hospital Case Book or Register of Patients." 10s. and 12s. 6d.**

**"The Furnishing and Appliances of a Cottage Hospital." 6d.**

**All these are published by the SCIENTIFIC PRESS, Ltd., and may be obtained through any bookseller, or direct from the publishers, 28 and 29 Southampton Street, Strand, London, W.C.**

### Useful Books for Nurses' Libraries.

### THE NURSING PROFESSION: HOW AND WHERE TO TRAIN.

Being a Guide to Training for the Calling of a Nurse.

Edited by Sir HENRY BURDETT, K.C.B.

Crown 8vo., 2|- net, 2/4 post free.

### A HANDBOOK FOR NURSES.

By J. K. WATSON, M.D., M.B., C.M.

New and Revised Edition.

Crown 8vo., profusely illustrated, cloth gilt.

5/- net, 5/4 post free.

### NURSING: ITS THEORY AND PRACTICE.

Being a complete Text-book of Medical, Surgical, and Monthly Nursing.

By PERCY C. LEWIS, M.D., M.R.C.S., L.S.A.

New Edition, Enlarged and Revised (17th thousand), profusely illustrated with over 100 new Cuts. Crown 8vo., cloth gilt.

3/6 post free.

### NURSING: ITS PRINCIPLES AND PRACTICE.

By ISABEL ADAMS HAMPTON.

New and Enlarged Edition.

Crown 8vo., illustrated, cloth gilt.

7/6 net, 7/10 post free.

### SURGICAL WARD-WORK AND NURSING.

By ALEXANDER MILES, M.D.Edin., C.M., F.R.C.S.E.

Second Edition, Enlarged and entirely Revised.

Demy 8vo., copiously illustrated, cloth gilt.

3/6 net, 3/10 post free.

### ELEMENTARY ANATOMY AND SURGERY FOR NURSES.

By WILLIAM McADAM ECCLES, M.S.Lond., M.B., F.R.C.S., L.R.C.P.

Crown 8vo., profusely illustrated,

Cloth. 2/6 post free.

### ELEMENTARY PHYSIOLOGY FOR NURSES.

By C. F. MARSHALL, M.D., B.Sc., F.R.C.S.

Crown 8vo., illustrated.

Cloth. 2/- post free.

### THE NURSE'S DICTIONARY OF MEDICAL TERMS AND NURSING TREATMENT.

Compiled for the Use of Nurses.

By HONNOR MORTEN.

Fourth and Revised Edition (50th thousand). Demy 16mo. (suitable for the apron pocket).

Bound in cloth boards, 160 pp., 2/- post free.

In handsome leather gilt, 2/6 net, 2/8 post free.

### HOSPITAL SISTERS AND THEIR DUTIES.

By EVA C. S. LÜCKES, Matron, London Hospital.

Third Edition, enlarged and partly re-written.

Crown 8vo., about 200 pp. cloth gilt.

2|6 post free.

### OPHTHALMIC NURSING.

By SYDNEY STEPHENSON, M.B., F.R.C.S.E.

New and Revised Edition.

Crown 8vo., profusely illustrated with Original Drawings, List of Instruments required, and a Glossary of Terms. Cloth.

3/6 net, 3/10 post free.

### ART OF MASSAGE.

By Mrs. CREIGHTON HALE.

Second Edition.

Demy 8vo., 150 pp., profusely illustrated with over 70 Special Cuts.

Cloth gilt,

6/- post free.

### A PRACTICAL HANDBOOK OF MIDWIFERY.

By FRANCIS W. HAULTAIN, M.D.

Ready Shortly.

New and Revised Edition.

Profusely illustrated with Original Cuts, Tables, &c.

Handsomely bound in leather.

6/- net, 6/3 post free.

*Of all Booksellers, or of*

THE SCIENTIFIC PRESS, LTD., 28 & 29 Southampton Street, Strand, London, W.C.

